# Comparative Transcriptome Profiling of an SV40-Transformed Human Fibroblast (MRC5CVI) and Its Untransformed Counterpart (MRC-5) in Response to UVB Irradiation

**DOI:** 10.1371/journal.pone.0073311

**Published:** 2013-09-03

**Authors:** Cheng-Wei Chang, Chaang-Ray Chen, Chao-Ying Huang, Wun-Yi Shu, Chi-Shiun Chiang, Ji-Hong Hong, Ian C. Hsu

**Affiliations:** 1 Department of Biomedical Engineering and Environmental Sciences, National Tsing Hua University, Hsinchu, Taiwan; 2 Institute of Statistics, National Tsing Hua University, Hsinchu, Taiwan; 3 Department of Radiation Oncology, Chang Gung Memorial Hospital, Taoyuan, Taiwan; 4 Department of Medical Imaging and Radiological Science, Chang Gung University, Taoyuan, Taiwan; Weizmann Institute of Science, Israel

## Abstract

Simian virus 40 (SV40) transforms cells through the suppression of tumor-suppressive responses by large T and small t antigens; studies on the effects of these two oncoproteins have greatly improved our knowledge of tumorigenesis. Large T antigen promotes cellular transformation by binding and inactivating p53 and pRb tumor suppressor proteins. Previous studies have shown that not all of the tumor-suppressive responses were inactivated in SV40-transformed cells; however, the underlying cause is not fully studied. In this study, we investigated the UVB-responsive transcriptome of an SV40-transformed fibroblast (MRC5CVI) and that of its untransformed counterpart (MRC-5). We found that, in response to UVB irradiation, MRC-5 and MRC5CVI commonly up-regulated the expression of oxidative phosphorylation genes. MRC-5 up-regulated the expressions of chromosome condensation, DNA repair, cell cycle arrest, and apoptotic genes, but MRC5CVI did not. Further cell death assays indicated that MRC5CVI was more sensitive than MRC-5 to UVB-induced cell death with increased caspase-3 activation; combining with the transcriptomic results suggested that MRC5CVI may undergo UVB-induced cell death through mechanisms other than transcriptional regulation. Our study provides a further understanding of the effects of SV40 transformation on cellular stress responses, and emphasizes the value of SV40-transformed cells in the researches of sensitizing neoplastic cells to radiations.

## Introduction

Simian virus 40 (SV40) is a polyomavirus that productively infects its natural host, *Rhesus macaque*, and more importantly, has the ability to neoplastically transform several types of nonpermissive cells and induce tumors in newborn hamsters [1]. Previous studies have established that the expressions of two SV40-encoded oncoproteins, the large T antigen and the small t antigen, are important for transformation by SV40. The large T antigen exerts its transformational ability by binding to p53 and pRb of host cells, thereby inhibiting p53-dependent tumor-suppressive responses and abrogating pRb-dependent cell cycle arrest at G1/S transition [2]–[3]. Recent studies have also shown still other factors that interact with the large T antigen and may play transformation roles [2], [4]–[5]. The small t antigen inhibits the protein phosphastase PP2A family members to perturb the PI3K/Akt pathway and c-Myc [6]–[7], and enhances the expression of miR-27A to facilitate malignant transformation [8]. These studies have significantly improved our understanding of the molecular mechanisms of tumorigenesis.

The tumor suppressor gene p53 plays a crucial role in response to stresses to maintain genome stability and prevent tumorigenesis, mainly through regulating the transcription of its target genes to initiate various tumor-suppressive responses such as cell cycle arrest, DNA repair, apoptosis, and senescence [9]–[12]. Recent studies have suggested that the regulation of energy metabolism is an additional tumor-suppressive function of p53 [13]–[15]. Under oncogenic stress, p53 directs cells to utilize mitochondrial oxidative phosphorylation rather than aerobic glycolysis that favors oncogenic process [16]–[17]. We previously showed that UVB irradiation induces the transactivation of genes functioning in oxidative phosphorylation in an untransformed human lung fibroblast [18]; other studies have also shown oxidative phosphorylation to be a major radiation-responsive pathway [19]–[21]. Because the large T antigen impedes p53 in SV40-transformed cells, and p53 regulates oxidative phosphorylation, this tumor-suppressive response might be considered inactive in SV40-transformed cells.

Although the binding of large T antigen to p53 suggests the subsequent abrogation of p53-dependent responses, previous studies have shown that SV40-transformed cells still have certain p53 responses [22]–[23]. In SV40-transformed cells, *CDKN1A* expression (which encodes protein p21) was induced in concert with the nuclear accumulation of active p53 in response to DNA damage [22]. Loss of p53 function contributes to chemoresistance in certain tumor types [10]; however, previous studies have reported that SV40-transformed cells are sensitive to DNA damage stresses. For example, SV40-transformed cells showed reduced expression of transcriptional target of p53, but these cells exhibited increased apoptosis to ionizing radiation relative to their untransformed counterparts [23], and the expression of SV40 large T antigen increased apoptosis and reduced clonogenic cell survival to UVC irradiation [24]. However the underlying cause of these observations is not fully studied.

In this study, we analyzed the gene expression profiles of an SV40-transformed human fibroblast (MRC5CVI) after UVB irradiation, as well as that of its untransformed counterpart (MRC-5). We applied cell cycle analysis and cell death assays to validate our findings of the trascriptomic study. Our investigations showed that MRC5CVI failed to regulate the expression of chromosome condensation, DNA repair, cell cycle arrest, and apoptotic genes, but not oxidative phosphorylation genes in response to UVB irradiation. Further cell death assays indicated that MRC5CVI was more sensitive than MRC-5 to UVB-induced cell death with increased caspase-3 activation; combining with the transcriptomic results suggested that MRC5CVI may undergo UVB-induced cell death through mechanisms other than transcriptional regulation.

## Results

### Overview of Microarray Data Revealed Similar and Dissimilar Gene Expression Responses to UVB Irradiation between MRC-5 and MRC5CVI

We performed a loop-designed microarray experiment to examine the time series gene expressions of MRC5CVI after a 600 J/m^2^ UVB irradiation (detailed in Figure S1). We reanalyzed the microarray data of our previous study for comparison, which examined gene expressions of MRC-5 with the same dose of UVB irradiation and an identical microarray design [18]. The microarray experiments for both cell types were carried out by the same platform and with the same loop-design and data-processing procedures to minimize possible experimental and data-processing bias. We identified 489 and 739 UVB-regulated genes in MRC-5 and MRC5CVI respectively (Table 1). For each cell type, UVB-regulated genes were grouped by *k*-means clustering with *k* = 6 and 1,000 iterations. The *k* = 6 criterion was chosen based on the observation of six major clusters by applying hierarchical clustering methods. By hierarchical clustering methods, a few genes will occasionally be missed from the six major clusters during the functionally enrichment analysis. To have an overview of cellular responses according to all the significantly regulated genes, we chose k-means clustering with k = 6 to avoid missing any genes.

**Table 1:** Number of UVB-regulated genes in MRC-5 and MRC5CVI.

	MRC-5	MRC5CVI	Union
Significant* at 4 h	154	168	272
Significant at 8 h	178	207	331
Significant at 16 h	180	376	472
Significant at 24 h	310	548	702
Significant at ≧1 time-points	489	739	967

* significant: fold-change >1.5 and Bonferroni-corrected *P*-value <0.05.

**Figure 1:**
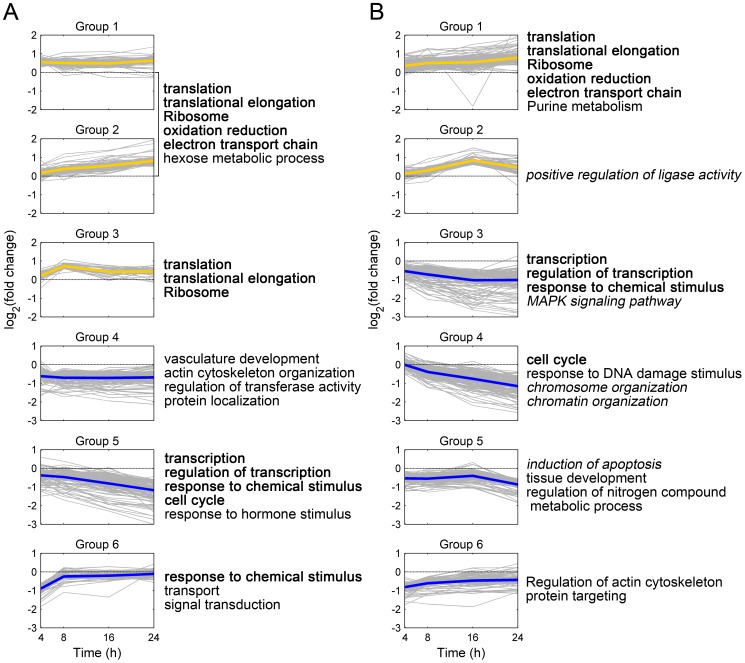
Clustering and functional enrichment analysis of UVB-regulated genes. UVB irradiation altered the expression of 489 and 739 genes in untransformed human fibroblast MRC-5 and its SV40-transformed counterpart MRC5CVI, respectively (Table 1). Clustering the UVB-regulated genes by *k*-means resulted in (A) 3 up-regulated groups and 3 down-regulated groups for MRC-5; (B) 2 up-regulated groups and 4 down-regulated groups for MRC5CVI. The colored thick line in each group plot represents the mean expression of genes in the group, with yellow indicating up-regulation and blue indicating down-regulation. The *x*-axis represents the time points after UVB irradiation, and the *y*-axis represents the log_2_-transformed fold change of gene expression. Groups of genes were further examined with functional enrichment analysis with annotation of Gene Ontology and the KEGG pathway. Representatively enriched functions are listed next to group plots. Bold functions indicate functions common to MRC-5 and MRC5CVI, and italic functions indicate functions unique to MRC5CVI.

The gene groups were then examined by functional enrichment analysis. Significant functions of up-regulated gene groups common to MRC-5 and MRC5CVI are mainly related to two functional categories, translation and oxidative phosphorylation (Figure 1A, Groups 1 to 3 for MRC-5; Figure 1B, Group 1 for MRC5CVI). Examining the expression patterns of oxidative phosphorylation genes revealed excellent concordant regulation of these genes in MRC-5 and MRC5CVI (Figure S2). Significant functions of down-regulated gene groups common to both cell types includes transcription, regulation of transcription, cell cycle, and response to chemical stimulus (Figure 1A, Groups 5 and 6 for MRC-5; Figure 1B, Groups 3 and 4 for MRC5CVI). For the dissimilar part, MRC5CVI up-regulated the genes related to ligase activity (Figure 1B, Group 2) and down-regulated the genes related to chromosome organization, the MAPK signaling pathway, and the induction of apoptosis in response to UVB irradiation (Figure 1B, Group 3 to 5). The gene groups of MRC-5 did not significantly enrich these functions.

### The Most Discrepant genes of MRC-5 and MRC5CVI Indicated their Regulatory Dissimilarities in Chromosome Condensation, DNA Repair, Cell Cycle Arrest, and Apoptosis

**Figure 2:**
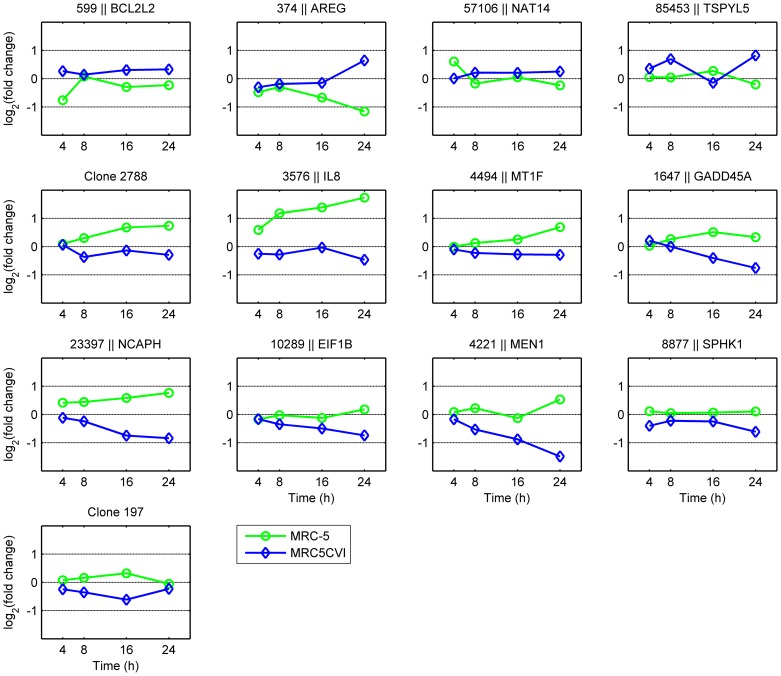
Expression patterns of the most discrepant genes between MRC-5 and MRC5CVI. These 13 genes were identified as the most discrepant genes between two cell types in response to UVB irradiation. The title in each subplot indicates the Entrez gene ID and gene name; clones cannot be matched to any Entrez gene ID labeled with their clone ID. The green line with the open circle indicates the gene expression pattern of MRC-5, whereas the blue line with the open diamond indicates that of MRC5CVI. The *x-*axis represents the time points after UVB irradiation, and the *y-*axis represents the log_2_-transformed fold change of gene expression.

**Figure 3:**
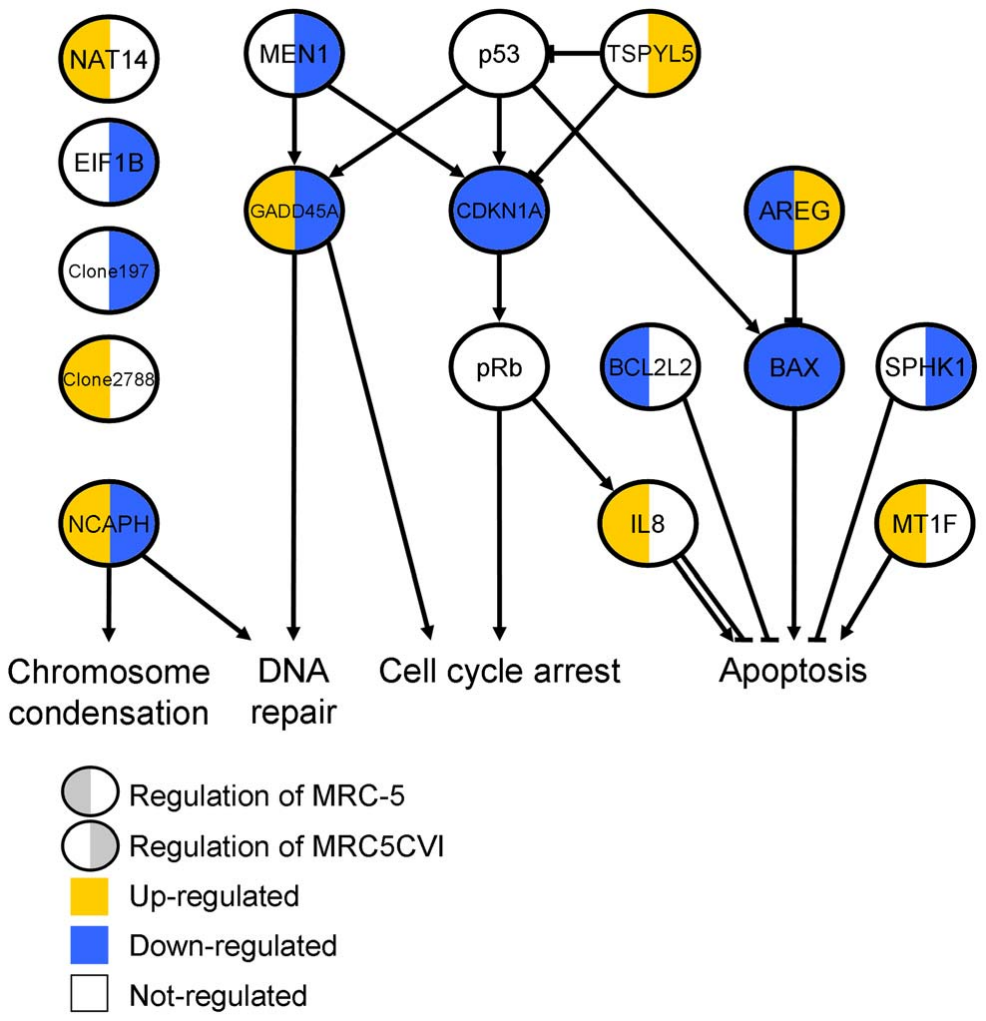
A literature-based model of the most discrepant genes. A literature search shows that the most discrepant genes are related to chromosome condensation, DNA repair, cell cycle arrest, and apoptosis (these studies are listed in Text S1). The p53/*CDKN1A*/pRb pathway and *BAX* are included to help clarify the results.

Combining 489 UVB-regulated genes in MRC-5 and 739 UVB-regulated genes in MRC5CVI resulted in 967 genes (Table 1). By filtering the expression patterns of the 967 genes between the two cell types with a criterion (Pearson correlation smaller than −0.519; detail in **Materials and Methods** - **Identification of the most discrepant genes between the two cell types**), 13 genes were identified as the most discrepant genes in response to UVB irradiation. The differential expression patterns of these 13 genes are shown in Figure 2. These 13 genes are related to chromosome condensation, DNA repair, cell cycle arrest, and apoptosis (Figure 3; related literatures are listed in Text S1). Previously we have validated our microarray system by RT-PCR with randomly selected genes, and the results were generally good (with R^2^ ranged from 0.86 to 0.94) [18], [25]–[26]. To verify the significant gene expression patterns in this study, we further performed RT-PCR analysis for certain UVB-regulated genes, including the transcriptional targets of p53 (*GADD45A* and *CDKN1A*), one antioxidant gene (*GPX1*), and three of the most discrepant genes (*MEN1*, *NCAPH*, and *IL8*). The RT-PCR results validated the microarray data of both cell types (R^2^ = 0.82 for MRC-5 and R^2^ = 0.88 for MRC5CVI; see Figure S3).

### UVB Induced G2/M Arrest in MRC-5, but Neither G1/S nor G2/M Arrest in MRC5CVI

**Figure 4:**
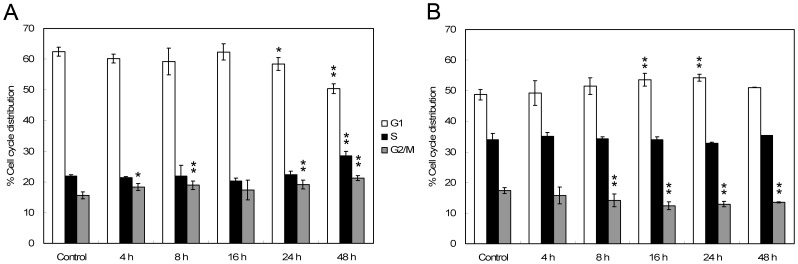
Cell cycle analysis of MRC-5 and MRC5CVI. Panel (A), results of MRC-5; panel (B), results of MRC5CVI. Pairwise statistical comparisons of the corresponding cell cycle phase between the time point and control were performed using Student’s *t* test (*, *P*<0.05; **, *P*<0.01). Error bars show the standard deviation (n = 3).

The protein encoded by the gene *NCAPH* plays a role in chromosome condensation [27]–[28], and is important in regulating mitotic cell death [29]. Both *NCAPH* and *GADD45A* are important in regulating DNA repair [30]–[32], thus their discrepant expressions suggest the difference in repair capability between the two cell types (Figure 3). *GADD45A* also plays a crucial role in regulating G2/M arrest [32]–[34]. The RT-PCR results validated the discrepant regulation of *GADD45A* between the two cell types, as well as the consistent down-regulation of *CDKN1A* in both cell types (Figure S4). In addition to the regulation by p53, the expression of *MEN1* [35]–[36] and *TSPYL5* [37]–[38] may play superior roles in regulating *GADD45A* and *CDKN1A* expressions in MRC5CVI. The discrepant regulation of *GADD45A* between MRC-5 and MRC5CV1 suggests their differences in regulating G2/M arrest in response to UVB irradiation. We then performed cell cycle analysis and found the increments of G2/M population for MRC-5 (Figure 4A), whereas only a gradual decrease of G2/M cells for MRC5CVI after UVB irradiation (Figure 4B). The consistent down-regulation of *CDKN1A* implies that both cell types may not regulate G1/S arrest. The cell cycle result confirmed that G1/S arrest was abrogated in both cell types in response to UVB irradiation.

### MRC5CVI was more Sensitive to UVB-induced Cell Death than MRC-5

**Figure 5:**
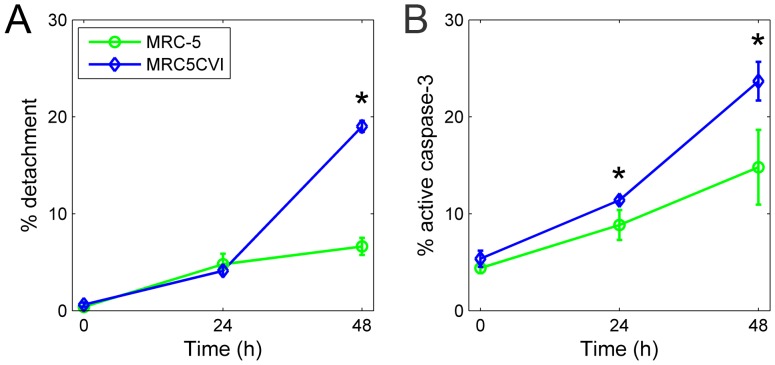
Examination of cell death after UVB irradiation by percent detachment and active caspase-3 staining. Panel (A), results of percent detachment; panel (B), results of active caspase-3 staining. Pairwise statistical comparisons between the time point of two cell types were performed using Student’s *t* test (*, *P*<0.05). Error bars show the standard deviation (n = 3).

Among the most discrepant genes between MRC-5 and MRC5CVI, five are functionally involved in apoptotic regulation (Figure 3). According to their roles in regulating apoptosis, their expressions favor a UVB-induced apoptotic response in MRC-5 cells, whereas only the significant down-regulation of *SPHK1* favors the pro-apoptotic status of MRC5CVI in response to UVB irradiation (detailed in the **Discussion** section). To examine the cell death response of both cell types after UVB irradiation, we performed two assays, as follows: estimating the percentage of cell detachment and active caspase-3 staining. Percent detachment was calculated as the number of cells in the medium compared to the total cells. Previous studies have used percent detachment as a quantitative estimate of cell death in fibroblasts [24], [39], and the activation of caspase-3 represents a major feature of apoptosis [40]. The results of cell death assays indicated that both cell types underwent cell death with activated caspase-3 after UVB irradiation, with MRC5CVI being more sensitive than MRC-5 (Figure 5A and 5B).

## Discussion

**Figure 6:**
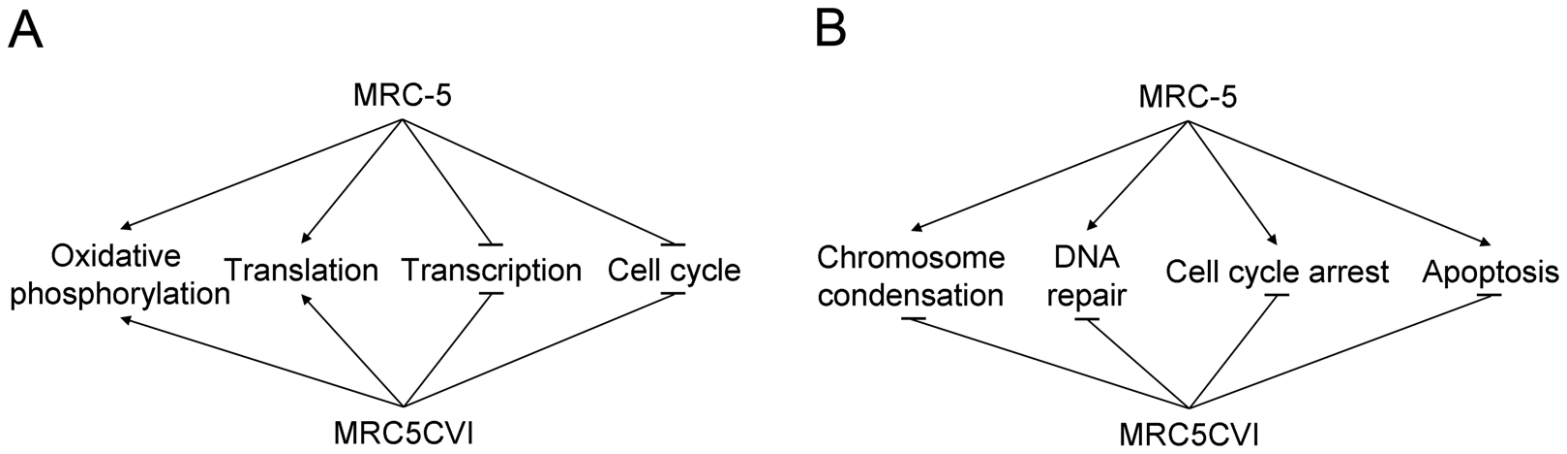
Summary of similar and dissimilar responses to UVB irradiation of MRC-5 and MRC5CVI. The UVB-induced responses are represented by functions that are (A) similarly or (B) dissimilarly regulated in MRC-5 and MRC5CVI. For example, the regulation of oxidative phosphorylation is similar, whereas the regulation of cell cycle arrest is dissimilar in the two cell types. The transcriptomic results suggest that the apoptosis response is suppressed in MRC5CVI; however, the results of the cell death assays indicate that MRC5CVI is more sensitive to UVB-induced cell death (see the **Discussion** section).

We investigated the UVB-responsive transcriptome of MRC5CVI and MRC-5. Based on the results of functional enrichment analysis, the literature review, cell cycle analysis, and cell death analysis, we summarized their responses to UVB irradiation into similar and dissimilar regulated functions (Figure 6A and 6B, respectively). In response to UVB irradiation, MRC5CVI was still able to up-regulate the expression of oxidative phosphorylation genes as MRC-5 was (Figure 6A and Figure S2). Furthermore, MRC5CVI failed to regulate the expression of chromosome condensation, DNA repair, cell cycle arrest, and apoptotic genes in human fibroblasts (Figure 6B). Our results of the cell death assays revealed that MRC5CVI was more sensitive to UVB-induced cell death than MRC-5, which is in agreement with the results of previous studies, which showed that SV40-transformed cells are more sensitive to radiation than their untransformed counterparts (see the following discussion) [23]–[24].

As discussed in our previous study, after UVB irradiation, the up-regulation of genes related to translation and ribosomal proteins in human fibroblasts suggested the need to renew UVB-damaged ribosomal proteins [18]. This corresponds to the result by the Casati and Walbot study, which showed that UVB irradiation induced direct ribosomal damage by crosslinking ribosomal proteins to RNA [41]. Furthermore, UV irradiation inflicted UV photoproducts on DNA, which impedes RNA polymerase and suppresses global transcription [42]. Our microarray data indicated that gene expression related to transcription was also down-regulated in both cell types (Figure 1), which may suggest a feedback control that retards the transcriptional process for DNA repair. The suppression of cell cycle genes implies the slowdown of cell cycle progression because of UVB-induced cellular damage. In addition, the genes of functions related to the actin cytoskeleton, development, protein targeting/localization, and response to chemical stimulus were down-regulated in both cell types (Figure 1). Gene regulation concerning translation, transcription, cell cycle, and the functions common to MRC-5 and MRC5CVI represents general cellular responses to UVB irradiation.

The up-regulation of oxidative phosphorylation is a major radiation-responsive pathway [19]–[21], which implies the need for energy to sustain cellular damage responses [18]. The up-regulation of oxidative phosphorylation was recently proposed to be a tumor-suppressive response under the control of p53 to reduce the generation of reactive oxygen species in response to stress [13]–[15]. However, our microarray data showed that genes related to oxidative phosphorylation were up-regulated in MRC5CVI as they were in MRC-5 (Figure S2), which suggests that the regulation of this response is intact in MRC5CVI. Thus, the regulation of oxidative phosphorylation may either be p53-independent in response to UVB irradiation or p53-dependent if large T antigen-bound p53 is still able to exert this regulation, but may play a minor role in the SV40 transformation process.

Persistent DNA lesions caused by UV irradiation activate p53 and the subsequent tumor-suppressive responses, including DNA repair, cell cycle arrest, and apoptosis [43]–[46]. We showed that among three of the p53 transcriptional targets (*GADD45A*, *CDKN1A*, and *BAX*), only *GADD45A* was up-regulated in MRC-5 in response to UVB irradiation (Figure S4; *BAX* is not in the probe sets of our microarray platform). The suppressed expressions of *CDKN1A* and *BAX* can be perceived with the results of previous studies that longer genes suffered more UV photoproducts that inflicted transcriptional blockage [24], [47]. The length of *GADD45A* is relatively shorter (3.2 kb) than that of *CDKN1A* (10.8 kb) and *BAX* (6.9 kb). Thus in MRC-5, *GADD45A* expression persists as a secondary defense to activate G2/M arrest and the DNA repair process when the first defense genes, such as *CDKN1A* and *BAX*, fail to be expressed after UVB irradiation. In MRC5CVI, the down-regulation of *GADD45A* after UVB irradiation is consistent with the impediment of the p53 function by large T antigen, and is related to the reduced DNA repair capacity and impaired G2/M arrest in SV40-transformed cells [48]–[50]. The concurrent down-regulation of chromosome condensation, DNA repair, and cell cycle arrest genes may cause genomic instability and lead to mitotic cell death for MRC5CVI. Therefore, the gradual decrease of G2/M cells after UVB irradiation for MRC5CVI (Figure 4B) may be the result of genome-unstable-caused cell death during the G2/M phase.

Sensitizing tumor cells to radiation is a crucial issue for radiobiologists [51]–[53]. The SV40-transformed cells may represent a sensitized neoplastic model, which enable us to investigate factors and mechanisms for sensitizing tumor cells. For example, previous studies have shown that the introduction of the SV40 T/t common polypeptide increased the sensitivity of HER2-overexpressing cancer cells to chemotherapy drugs, and these reports have suggested that this is attributed to the inhibition of Bcl-2, Bcl-XL, and ERK by the polypeptide [54]–[56]. Our results of the cell death assays showed that MRC5CVI was more sensitive to UVB-induced cell death than MRC-5 (Figure 5), despite the functional enrichment analysis of microarray data having indicated that genes related to the induction of apoptosis were down-regulated in MRC5CVI in response to UVB irradiation (Figure 1). In addition, the regulation of the most discrepant genes did not favor the apoptotic status of MRC5CVI (Figure 3). The protein product of the *AREG* sequesters the *BAX* protein in the cytoplasm to suppress the apoptotic process [57]. The *BCL2L2* of the Bcl-2 family is an important anti-apoptotic gene [58], and suppression of its expression sensitizes cells to anticancer drugs [59]–[60], whereas *MT1F* exerts its tumor-suppressive ability by inducing apoptosis [61]. Thus, the down-regulation of *AREG*, the early suppression of *BCL2L2*, and the up-regulation of *MT1F* expressions imply an early onset of apoptosis for MRC-5 in response to UVB irradiation, whereas all of these gene expressions do not suggest an apoptotic response in MRC5CVI (Figure 3). In addition, pro-apoptotic genes transactivated by p53 were either down-regulated (*BAX*) or not significantly regulated (*APAF1*, *BID*, *BAK1*, and *PMAIP1*) in MRC5CVI (Figure S4 and S5). Only the significant down-regulation of *SPHK1*, which is an anti-apoptotic gene that protects cells to UVB irradiation [62]–[63], may confer a pro-apoptotic phenotype to MRC5CVI in response to UVB irradiation. Thus, an overview of transcriptomic results suggests that the increased UVB-induced cell death of MRC5CVI is transcription-independent of the regulation of apoptotic genes. MRC5CVI may undergo cell death through mechanisms other than those in MRC-5. Several mechanisms might account for this possibility: (a) The large T antigen-bound p53 protein may still be able to induce cell death through a transcription-independent pathway. This hypothesis is supported by recent studies showing that physical interactions of p53 with various members of the Bcl-2 family provide a transcription-independent process of p53-mediated cell death (see reviews of [64]–[65]). (b) Down-regulation of chromosome condensation and the DNA repair genes after UVB irradiation in coordination with the lost control of cell cycle arrest may push MRC5CVI into a genome-unstable and pro-apoptotic status, which leads to mitotic cell death. The factor that initiates cell death progression under this status requires further exploration if it is not p53. (c) Down-regulation of *SPHK1* may be sufficient to sensitize MRC5CVI to UVB-induced cell death. If this is true, how SV40 transformation alters the expression of *SPHK1* is worth exploring, as well as whether this alternation applies to sensitivities in response to other genotoxic stresses. All of these hypotheses await further investigation, and the corresponding results may provide insights into the sensitization of neoplastic cells to radiation.

Our results may provide insights into the effects of SV40 transformation, and benefit further researches on those transformation-affected tumor-suppressive responses. We hope that this study could lead to more comprehensive investigations of the stress response of multiple independently derived SV40-transformed cell lines.

## Materials and Methods

### Cell Culture and UVB Irradiation

Normal human fibroblasts MRC-5 (CCL-171, purchased from American Type Culture Collection, Manassas; and BCRC-60023, purchased from Bioresource Collection and Research Center, Taiwan) were grown as previously described [18]. SV40-transformed human lung fibroblast MRC5CVI was a kind gift from Dr. C. Arlett (MRC Genome Damage and Stability Centre, University of Sussex, UK; formerly the MRC Cell Mutation Unit) to Dr. J.H. Hong with related publications [66]–[67]. MRC5CVI were grown in Dulbecco’s modified Eagle medium (Gibco-BRL, Grand Island, NY) supplemented with 10% heat-inactivated fetal bovine serum (HyClone, Logan, UT), 100 U/ml penicillin, 10 µg/ml streptomycin with 0.29 mg/ml glutamine (Gibco-BRL, Grand Island, NY) at 37°C in humidified air with 5% CO_2_. The UVB-light source was a 6-W UVB fluorescent tube (model Spectroline EB-160C, Spectronics Corporation, Westbury, NY) and was set up as described previously [18]. The UVB dose was measured by a photometer (model IL 1400A, International Light Inc., Newburyport, MA). Cells were seeded in 10 cm cell culture dishes and let grow to 40% confluence. Before UVB irradiation, the growth medium for each dish was collected in a centrifuge tube; cells were washed twice with PBS and then irradiated with 600 J/m^2^ UVB in an uncovered dish with a thin layer of PBS. An accompanied control dish of cells was treated identically for each time point, except for UVB radiation. After UVB irradiation, cells were replenished with the previously collected growth medium and incubated for 4, 8, 16, or 24 h before harvest. The culture and the UVB irradiation procedure for both cell types were carried out simultaneously with the same incubator and UVB-irradiating system to minimize possible technical bias.

### Microarray Analysis

Microarray fabrication, hybridization, and data preprocessing were performed as previously described [18]. Cells were harvested with TRIzol reagent (Invitrogen, Carlsbad, CA) to extract total RNA according to manufacturer protocols at scheduled time points. The total RNA was further purified by RNeasy (Quiagen, Valencia, CA). The quality of total RNA was assessed using the Agilent 2100 bioanalyzer with the RNA 6000 Nano LabChip kit (Agilent Technologies, Palo Alto, CA). Reverse transcription and microarray hybridization were conducted using SuperScript II (Invitrogen) and the 3DNA Array 50™ kit (Genisphere, Hatfield, PA) following manufacturer protocols. A total of 8 RNA samples (control and UVB irradiated samples at 4, 8, 16, or 24 h after treatment) were hybridized with 10 microarrays in a loop-design as previously described (Figure S1) [18]. Microarray data preprocessing, normalization, and statistical analysis were performed by a bioinformatics software suit: Tsing Hua Engine for Microarray Experiment (THEME [68]. Pin-wise lowess-normalized microarray data were further processed by using a log linear model as described previously [18], [68]. Differentially expressed cDNA clones for each time point were identified by applying criteria of the Bonferroni-adjusted F test *P*-value <0.05 and a fold change of at least 1.5. The microarray data of MRC5CVI was submitted to Gene Expression Omnibus (GEO, Series accession number GSE41319), and the microarray data of MRC-5 from our previous study was reanalyzed for comparison (Series accession number GSE7589) [18].

### Identification of the Most Discrepant genes between the two Cell Types

We identified 489 and 739 UVB-regulated genes in MRC-5 and MRC5CVI respectively (Table 1). Combining these genes resulted in 967 genes as the candidates for selecting the most discrepant genes between the two cell types in response to UVB irradiation. We first calculated the Pearson correlation of the expression patterns of each of the 967 genes between the two cell types. We then estimated the random correlation by randomly matching all of the genes in MRC-5 to the genes in MRC5CVI and calculated the random match correlations. After 100 iterations, the results converged toward a distribution of random correlation with a mean close to zero and a standard deviation of 0.519. Most of the Pearson correlations of the 967 genes are greater than 0.519 (583 out of 967 genes). By selecting genes with a Pearson correlation smaller than −0.519, 13 genes are identified as the most discrepant genes between the two cell types in response to UVB irradiation.

### Functional Annotation and Functional Enrichment Analysis

Functional annotation and functional enrichment analysis was accessed through DAVID (Database for Annotation, Visualization, and Integrated Discovery) [69]–[70], which performed a modified Fisher’s exact test (with EASE score as the *P*-value) to select over-represented functions. The Entrez gene ID was used as the gene-specific identifier for bioinformatics analysis. KEGG pathways or GO terms containing more than 5 genes and with an EASE score smaller than 0.05 were considered significant.

### Cell-cycle Analysis and Cell Death Assays

For cell-cycle analysis, 5×10^5^ cells were seeded in 10 cm cell culture dishes and allowed to grow for 24 h. At 4, 8, 16, 24, and 48 h after UVB irradiation, attached cells were trypsinized and collected with the cells in growth medium, washed in iced PBS, and then fixed with ethanol at −20°C overnight. Before analysis, cells were washed twice in iced PBS and stained with a 2 ml propidium iodide staining solution (20 µg/ml of propidium iodide, 200 µg/ml of RNase A, and 0.2% Triton X-100 in PBS) at 37°C for 30 min. Cell cycle analysis was performed by FACScan (BD Biosciences), and the result was analyzed with Modfit LT 3.2 software (Verity Software House, Inc.). For active caspase-3 staining, cells were UVB irradiated, collected, and fixed with ethanol at −20°C overnight. After fixation, cells were washed twice in iced PBS, and then followed the instruction of the FITC Active Caspase-3 Apoptosis Kit (BD Biosciences, cat#550480). The results were analyzed with WinMDI 2.8 software (Scripps, La Jolla, CA). For cell detachment analysis, at 24 and 48 h after UVB irradiation, detached cells were collected from the growth medium by centrifugation, whereas attached cells were washed with PBS, trypsinized, and collected by centrifugation separately. Cells were resuspended in 1 ml cold PBS and the cell numbers of detached and attached cells were determined by hemocytometer counting. The percent detachment was calculated as the number of detached cells over the total number of detached and attached cells.

### Real-Time PCR Assay

Real-time PCR (RT-PCR) assay was performed using Power SYBR Green Master Mix and ABI Prism 7300 Real-Time PCR System (Applied Biosystems, Foster City, CA). Gene specific primers were designed using the Primer-BLAST [71]; only primers without non-specific targets were used. All primers used in this study are provided in Table S1. Assays in triplicate were conducted using the following conditions: 2 min at 50°C, 10 min at 95°C and then 40 cycles of amplification (95 °C for 15 s, 60°C for 30 s, and 72°C for 45 s). The quantity of target mRNAs was normalized to that of a reference gene, *PPIA*.

## Supporting Information

Figure S1 **Flowchart of the design of UVB exposure and loop-designed microarray.** 4UV, 8UV, 16UV and 24UV denote samples that were harvested at 4, 8, 16 and 24 h after 600 J/m^2^ of UVB irradiation respectively. 4C, 8C, 16C and 24C denote the accompanied control samples. Each arrow indicates a microarray hybridization experiment. The arrowheads represent samples that were labeled with Cy5, and the tails represent samples that were labeled with Cy3. The microarray data of MRC5CVI was submitted to Gene Expression Omnibus (GEO, Series accession number GSE41319), and the microarray data of MRC-5****from our previous study was reanalyzed for comparison (Series accession number GSE7589).
(PDF)

Figure S2 **The expression patterns of genes related to oxidative phosphorylation.** The title in each subplot indicates the Entrez gene ID and gene name. The green line with the open circle indicates the gene expression pattern of MRC-5, whereas the blue line with the open diamond indicates that of MRC5CVI. The *x-*axis represents the time points after UVB irradiation, and the *y-*axis represents the log_2_-transformed fold change of gene expression.
(PDF)

Figure S3 **Validation of microarray data by RT-PCR.** RT-PCR was performed to verify the microarray data across all time points. The genes selected were *GADD45A*, *CDKN1A*, *GPX1*, and *IL8* for MRC-5; *GADD45A*, *CDKN1A*, *GPX1*, *MEN1*, and *NCAPH* for MRC5CVI. The expression levels of a reference gene, *PPIA*, were used to normalize that of target genes. RT-PCR analysis of these genes verified the microarray data, with (A) R^2^ = 0.82 for MRC-5 and (B) R^2^ = 0.88 for MRC5CVI. The *x-*axis represents the log_2_-transformed fold change of gene expression of microarray data, and the *y-*axis represents that of RT-PCR data.
(PDF)

Figure S4 **Results of RT-PCR for three transcriptional targets of p53.** By applying RT-PCR, we examined the expressions of 3 genes (*GADD45A*, *CDKN1A*, and *BAX*; *BAX* is not in the probe sets of our microarray platform), which are transcriptionally regulated by p53. The title in each subplot indicates the Entrez gene ID and gene name. The green line with the open circle indicates the gene expression pattern of MRC-5, whereas the blue line with the open diamond indicates that of MRC5CVI. The solid line indicates the result of microarray, whereas the dashed line indicates the result of RT-PCR. The *x-*axis represents the time points after UVB irradiation, and the *y-*axis represents the log_2_-transformed fold change of gene expression.
(PDF)

Figure S5 **The expression patterns of** ***TP53*** **and the genes related to regulation of apoptosis.** The title in each subplot indicates the Entrez gene ID and gene name. The green line with the open circle indicates the gene expression pattern of MRC-5, whereas the blue line with the open diamond indicates that of MRC5CVI. The *x-*axis represents the time points after UVB irradiation, and the *y-*axis represents the log_2_-transformed fold change of gene expression.
(PDF)

Table S1 **Primer sequences for RT-PCR.**
(PDF)

Text S1 **Literature review of the most discrepant genes.**
(PDF)
