# Supplementary material for: Comparative Transcriptome Profiling of an SV40-Transformed Human Fibroblast (MRC5CVI) and Its Untransformed Counterpart (MRC-5) in Response to UVB Irradiation
Source: PLoS One. 2013 Sep 3;8(9):e73311. doi: 10.1371/journal.pone.0073311 (PMC3760899; doi:10.1371/journal.pone.0073311)
Supplement: Figure S1 — Flowchart of the design of UVB exposure and loop-designed microarray. 4UV, 8UV, 16UV and 24UV denote samples that were harvested at 4, 8, 16 and 24 h after 600 J/m2 of UVB irradiation respectively. 4C, 8C, 16C and 24C denote the accompanied control samples. Each arrow indicates a microarray hybridization experiment. The arrowheads represent samples that were labeled with Cy5, and the tails represent samples that were labeled with Cy3. The microarray data of MRC5CVI was submitted to Gene Expression Omnibus (GEO, Series accession number GSE41319), and the microarray data of MRC-5from our previous study was reanalyzed for comparison (Series accession number GSE7589). (PDF) [file pone.0073311.s001.pdf]

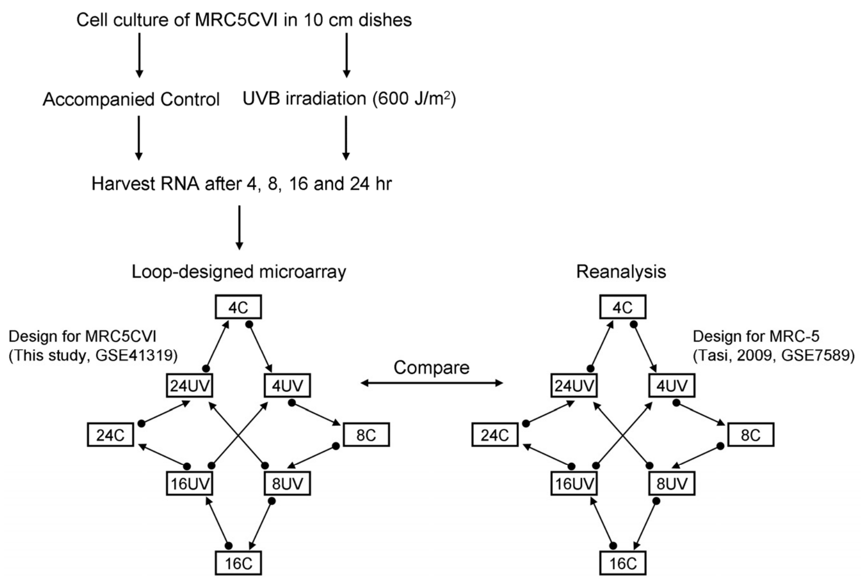


**Figure S1**

**Flowchart of the design of UVB exposure and loop-designed microarray.** 4UV, 8UV, 16UV and 24UV denote samples that were harvested at 4, 8, 16 and 24 h after 600 J/m$^2$ of UVB irradiation respectively. 4C, 8C, 16C and 24C denote the accompanied control samples. Each arrow indicates a microarray hybridization experiment. The arrowheads represent samples that were labeled with Cy5, and the tails represent samples that were labeled with Cy3. The microarray data of MRC5CVI was submitted to Gene Expression Omnibus (GEO, Series accession number GSE41319), and the microarray data of MRC-5 from our previous study was reanalyzed for comparison (Series accession number GSE7589).
