# Supplementary material for: Comparative Transcriptome Profiling of an SV40-Transformed Human Fibroblast (MRC5CVI) and Its Untransformed Counterpart (MRC-5) in Response to UVB Irradiation
Source: PLoS One. 2013 Sep 3;8(9):e73311. doi: 10.1371/journal.pone.0073311 (PMC3760899; doi:10.1371/journal.pone.0073311)
Supplement: Figure S3 — Validation of microarray data by RT-PCR. RT-PCR was performed to verify the microarray data across all time points. The genes selected were GADD45A, CDKN1A, GPX1, and IL8 for MRC-5; GADD45A, CDKN1A, GPX1, MEN1, and NCAPH for MRC5CVI. The expression levels of a reference gene, PPIA, were used to normalize that of target genes. RT-PCR analysis of these genes verified the microarray data, with (A) R2 = 0.82 for MRC-5 and (B) R2 = 0.88 for MRC5CVI. The x-axis represents the log2-transformed fold change of gene expression of microarray data, and the y-axis represents that of RT-PCR data. (PDF) [file pone.0073311.s003.pdf]

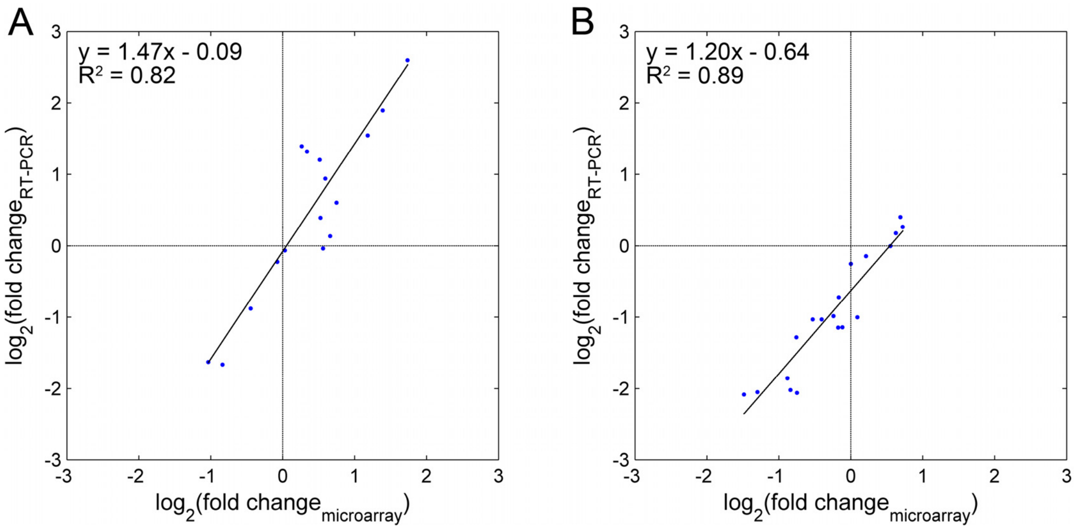

Figure S3

Validation of microarray data by RT-PCR. RT-PCR was performed to verify the microarray data across all time points. The genes selected were *GADD45A*, *CDKN1A*, *GPX1*, and *IL8* for MRC-5; *GADD45A*, *CDKN1A*, *GPX1*, *MEN1*, and *NCAPH* for MRC5CVI. The expression levels of a reference gene, *PPIA*, were used to normalize that of target genes. RT-PCR analysis of these genes verified the microarray data, with (A) R^2 = 0.82 for MRC-5 and (B) R^2 = 0.88 for MRC5CVI. The *x*-axis represents the $\log_2$-transformed fold change of gene expression of microarray data, and the *y*-axis represents that of RT-PCR data.
