# Supplementary material for: Comparative Transcriptome Profiling of an SV40-Transformed Human Fibroblast (MRC5CVI) and Its Untransformed Counterpart (MRC-5) in Response to UVB Irradiation
Source: PLoS One. 2013 Sep 3;8(9):e73311. doi: 10.1371/journal.pone.0073311 (PMC3760899; doi:10.1371/journal.pone.0073311)
Supplement: Figure S4 — Results of RT-PCR for three transcriptional targets of p53. By applying RT-PCR, we examined the expressions of 3 genes (GADD45A, CDKN1A, and BAX; BAX is not in the probe sets of our microarray platform), which are transcriptionally regulated by p53. The title in each subplot indicates the Entrez gene ID and gene name. The green line with the open circle indicates the gene expression pattern of MRC-5, whereas the blue line with the open diamond indicates that of MRC5CVI. The solid line indicates the result of microarray, whereas the dashed line indicates the result of RT-PCR. The x-axis represents the time points after UVB irradiation, and the y-axis represents the log2-transformed fold change of gene expression. (PDF) [file pone.0073311.s004.pdf]

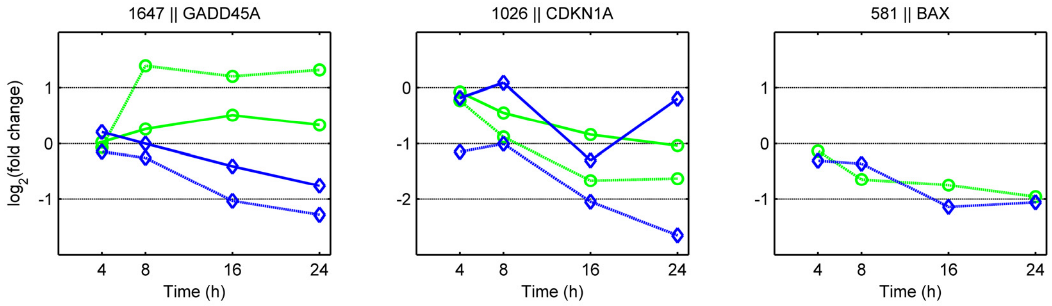

Figure S4

Results of RT-PCR for three transcriptional targets of p53. By applying RT-PCR, we examined the expressions of 3 genes (*GADD45A*, *CDKN1A*, and *BAX*; *BAX* is not in the probe sets of our microarray platform), which are transcriptionally regulated by p53. The title in each subplot indicates the Entrez gene ID and gene name. The green line with the open circle indicates the gene expression pattern of MRC-5, whereas the blue line with the open diamond indicates that of MRC5CVI. The solid line indicates the result of microarray, whereas the dashed line indicates the result of RT-PCR. The *x*-axis represents the time points after UVB irradiation, and the *y*-axis represents the $\log_2$-transformed fold change of gene expression.
