# Supplementary material for: Comparative Transcriptome Profiling of an SV40-Transformed Human Fibroblast (MRC5CVI) and Its Untransformed Counterpart (MRC-5) in Response to UVB Irradiation
Source: PLoS One. 2013 Sep 3;8(9):e73311. doi: 10.1371/journal.pone.0073311 (PMC3760899; doi:10.1371/journal.pone.0073311)
Supplement: Figure S5 — The expression patterns of TP53 and the genes related to regulation of apoptosis. The title in each subplot indicates the Entrez gene ID and gene name. The green line with the open circle indicates the gene expression pattern of MRC-5, whereas the blue line with the open diamond indicates that of MRC5CVI. The x-axis represents the time points after UVB irradiation, and the y-axis represents the log2-transformed fold change of gene expression. (PDF) [file pone.0073311.s005.pdf]

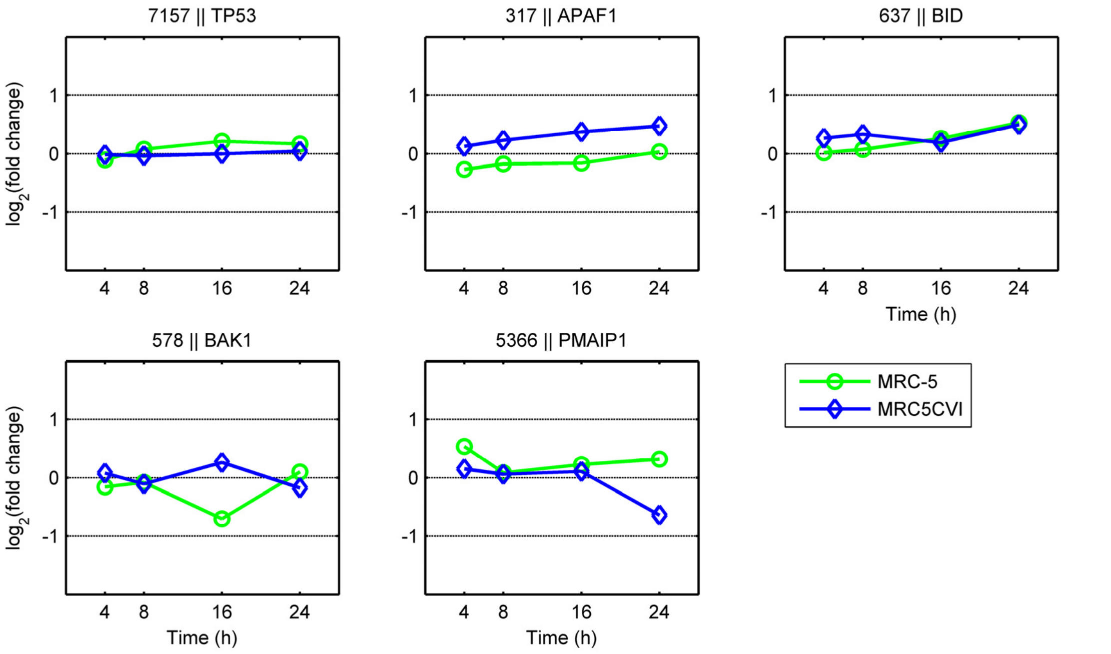

Figure S5

The expression patterns of *TP53* and the genes related to regulation of apoptosis. The title in each subplot indicates the Entrez gene ID and gene name. The green line with the open circle indicates the gene expression pattern of MRC-5, whereas the blue line with the open diamond indicates that of MRC5CVI. The *x*-axis represents the time points after UVB irradiation, and the *y*-axis represents the $\log_2$-transformed fold change of gene expression.
