# Supplementary material for: Comparative Transcriptome Profiling of an SV40-Transformed Human Fibroblast (MRC5CVI) and Its Untransformed Counterpart (MRC-5) in Response to UVB Irradiation
Source: PLoS One. 2013 Sep 3;8(9):e73311. doi: 10.1371/journal.pone.0073311 (PMC3760899; doi:10.1371/journal.pone.0073311)
Supplement: Table S1 — Primer sequences for RT-PCR. (PDF) [file pone.0073311.s006.pdf]

Table S1 - Primer sequences for RT-PCR

Entrez Gene ID	Gene Name	Accession #	Forward primer sequence (5' to 3')	Reverse primer sequence (5' to 3')
1647	GADD45A	NM_001199741.1	TCTCCCTGAACGGTGATGGC	CAGCCCCTTGGCATCAGTTT
1026	CDKN1A	NM_001220778.1	TTCTACCACTCCAAACGCCG	GCAGAAGATGTAGAGCGGGC
581	BAX	NM_138761.3	CTGAGCGAGTGTCTCAAGCG	CCCCAGTTGAAGTTGCCGTC
2876	GPX1	NM_000581.2	GTCGGTGTATGCCTTCTCGG	TCAGAGGGACGCCACATTCT
3576	IL8	NM_000584.3	ACACTGCGCCAACACAGAAA	AAACTTCTCCACAACCCTCTGC
4221	MEN1	NM_130801.2	CGCCCTCTTCCTGACGAAAC	TCAGGCTCTTGTCACCCACT
23397	NCAPH	NM_015341.3	TCACCTCAAACCAGGCACCA	CAGGGCAAAAGTTGGAGGTGT
5478	PPIA	NM_021130.3	TACGGGTCCTGGCATCTTGT	GCTTGCCATCCAACCACTCA
