# Supplementary material for: Comparative Transcriptome Profiling of an SV40-Transformed Human Fibroblast (MRC5CVI) and Its Untransformed Counterpart (MRC-5) in Response to UVB Irradiation
Source: PLoS One. 2013 Sep 3;8(9):e73311. doi: 10.1371/journal.pone.0073311 (PMC3760899; doi:10.1371/journal.pone.0073311)
Supplement: Text S1 — Literature review of the most discrepant genes. (PDF) [file pone.0073311.s007.pdf]

Text S1 Literature review of the most discrepant genes.

The table and the following references describe the regulations in Figure 3 of the main text. The + indicates positive-regulation, where as the - indicates negative-regulation. Reference indicates the corresponding study describing the regulation. The **Related function** column describes either the effects of the regulation of up-stream gene on down-stream gene, or the functions of the up-stream gene. For example, *GADD45A* (down-stream gene) is positive-regulated by *MEN1* (up-stream gene) according to the result of Ref. 7, and this regulation is related to the positive-regulation of DNA repair and apoptosis according to the result of Ref. 8 and Ref. 9 respectively.

**Up-stream gene**		**Down-stream gene**			**Related function**				
Entrez ID	Gene Name	Entrez ID	Gene Name	regulation	DNA repair	cell cycle arrest	apoptosis	chromosome condensation	sensitivity
1647	GADD45A				+ [1,2,3]	+ [1,4,5,6]			- [1,2]
4221	MEN1	1647	GADD45A	+ [7]	+ [8]		+ [9]		
4221	MEN1	1026	CDKN1A	+ [7,10]					
23397	NCAPH				+ [11]			+ [12,13,14]	
3576	IL8						+ [15] - [16]		- [16]
5925	RB1	3576	IL8	+ [17]					
599	BCL2L2						- [18,19,20]		- [20]
8877	SPHK1						- [21,22]		- [21]
374	AREG	581	BAX	- [23]			- [23]		
85453	TSPYL5	7157	TP53	- [24]					
85453	TSPYL5	1026	CDKN1A	- [25]					
4494	MT1F						+ [26]		

Reference

1. Maeda T, Hanna AN, Sim AB, Chua PP, Chong MT, et al. (2002) GADD45 regulates G2/M arrest, DNA repair, and cell death in keratinocytes following ultraviolet exposure. J Invest Dermatol 119: 22-26.
2. Smith ML, Ford JM, Hollander MC, Bortnick RA, Amundson SA, et al. (2000) p53-mediated DNA repair responses to UV radiation: studies of mouse cells lacking p53, p21, and/or gadd45 genes. Mol Cell Biol 20: 3705-3714.
3. Maeda T, Espino RA, Chomey EG, Luong L, Bano A, et al. (2005) Loss of p21WAF1/Cip1 in Gadd45-deficient keratinocytes restores DNA repair capacity. Carcinogenesis 26: 1804-1810.

4. Jin S, Tong T, Fan W, Fan F, Antinore MJ, et al. (2002) GADD45-induced cell cycle G2-M arrest associates with altered subcellular distribution of cyclin B1 and is independent of p38 kinase activity. Oncogene 21: 8696-8704.
5. Hollander MC, Sheikh MS, Bulavin DV, Lundgren K, Augeri-Henmueller L, et al. (1999) Genomic instability in Gadd45a-deficient mice. Nat Genet 23: 176-184.
6. Marrot L, Belaidi JP, Jones C, Perez P, Meunier JR (2005) Molecular responses to stress induced in normal human caucasian melanocytes in culture by exposure to simulated solar UV. Photochem Photobiol 81: 367-375.
7. Francis J, Lin W, Rozenblatt-Rosen O, Meyerson M (2011) The menin tumor suppressor protein is phosphorylated in response to DNA damage. PLoS One 6: e16119.
8. Jin S, Mao H, Schnepp RW, Sykes SM, Silva AC, et al. (2003) Menin associates with FANCD2, a protein involved in repair of DNA damage. Cancer Res 63: 4204-4210.
9. Schnepp RW, Mao H, Sykes SM, Zong WX, Silva A, et al. (2004) Menin induces apoptosis in murine embryonic fibroblasts. J Biol Chem 279: 10685-10691.
10. Kottemann MC, Bale AE (2009) Characterization of DNA damage-dependent cell cycle checkpoints in a menin-deficient model. DNA Repair (Amst) 8: 944-952.
11. Heale JT, Ball AR, Jr., Schmiesing JA, Kim JS, Kong X, et al. (2006) Condensin I interacts with the PARP-1-XRCC1 complex and functions in DNA single-strand break repair. Mol Cell 21: 837-848.
12. Ono T, Losada A, Hirano M, Myers MP, Neuwald AF, et al. (2003) Differential contributions of condensin I and condensin II to mitotic chromosome architecture in vertebrate cells. Cell 115: 109-121.
13. Tada K, Susumu H, Sakuno T, Watanabe Y (2011) Condensin association with histone H2A shapes mitotic chromosomes. Nature 474: 477-483.
14. Lai SK, Wong CH, Lee YP, Li HY (2011) Caspase-3-mediated degradation of condensin Cap-H regulates mitotic cell death. Cell Death Differ 18: 996-1004.
15. Balasubramanian A, Munshi N, Koziel MJ, Hu Z, Liang TJ, et al. (2005) Structural proteins of Hepatitis C virus induce interleukin 8 production and apoptosis in human endothelial cells. J Gen Virol 86: 3291-3301.
16. Efimova EV, Liang H, Pitroda SP, Labay E, Darga TE, et al. (2009) Radioresistance of Stat1 over-expressing tumour cells is associated with suppressed apoptotic response to cytotoxic agents and increased IL6-IL8 signalling. Int J Radiat Biol 85: 421-431.
17. Zhang H, Shepherd AT, Eason DD, Wei S, Diaz JI, et al. (1999) Retinoblastoma protein expression leads to reduced Oct-1 DNA binding activity and enhances interleukin-8 expression. Cell Growth Differ 10: 457-465.
18. Crawford M, Batte K, Yu L, Wu X, Nuovo GJ, et al. (2009) MicroRNA 133B targets pro-survival molecules MCL-1 and BCL2L2 in lung cancer. Biochem Biophys Res Commun 388: 483-489.

19. Uittenbogaard M, Baxter KK, Chiaramello A (2009) Cloning and characterization of the 5'UTR of the rat anti-apoptotic Bcl-w gene. Biochem Biophys Res Commun 389: 657-662.
20. Yang X, Yin J, Yu J, Xiang Q, Liu Y, et al. (2012) miRNA-195 sensitizes human hepatocellular carcinoma cells to 5-FU by targeting BCL-w. Oncol Rep 27: 250-257.
21. Song L, Xiong H, Li J, Liao W, Wang L, et al. (2011) Sphingosine kinase-1 enhances resistance to apoptosis through activation of PI3K/Akt/NF-kappaB pathway in human non-small cell lung cancer. Clin Cancer Res 17: 1839-1849.
22. Uchida Y, Houben E, Park K, Douangpanya S, Lee YM, et al. (2010) Hydrolytic pathway protects against ceramide-induced apoptosis in keratinocytes exposed to UVB. J Invest Dermatol 130: 2472-2480.
23. Busser B, Sancey L, Josserand V, Niang C, Favrot MC, et al. (2010) Amphiregulin promotes BAX inhibition and resistance to gefitinib in non-small-cell lung cancers. Mol Ther 18: 528-535.
24. Epping MT, Meijer LA, Krijgsman O, Bos JL, Pandolfi PP, et al. (2011) TSPYL5 suppresses p53 levels and function by physical interaction with USP7. Nat Cell Biol 13: 102-108.
25. Kim EJ, Lee SY, Kim TR, Choi SI, Cho EW, et al. (2010) TSPYL5 is involved in cell growth and the resistance to radiation in A549 cells via the regulation of p21(WAF1/Cip1) and PTEN/AKT pathway. Biochem Biophys Res Commun 392: 448-453.
26. Yan DW, Fan JW, Yu ZH, Li MX, Wen YG, et al. (2012) Downregulation of Metallothionein 1F, a putative oncosuppressor, by loss of heterozygosity in colon cancer tissue. Biochim Biophys Acta 1822: 918-926.
